# Spatiotemporal patterns of population in mainland China, 1990 to 2010

**DOI:** 10.1038/sdata.2016.5

**Published:** 2016-02-16

**Authors:** Andrea E. Gaughan, Forrest R. Stevens, Zhuojie Huang, Jeremiah J. Nieves, Alessandro Sorichetta, Shengjie Lai, Xinyue Ye, Catherine Linard, Graeme M. Hornby, Simon I. Hay, Hongjie Yu, Andrew J. Tatem

**Affiliations:** 1 Department of Geography and Geosciences, University of Louisville, Louisville, Kentucky, 40292 USA; 2 Division of Infectious Diseases, Key Laboratory of Surveillance and Early-warning on Infectious Disease, Chinese Center for Disease Control and Prevention, 155 Changbai Road, Changping District, Beijing 102206, China; 3 Geography and Environment, University of Southampton SO17 1BJ, UK; 4 Institute for Life Sciences, University of Southampton SO17 1BJ, UK; 5 Flowminder Foundation, Stiftelsen Flowminder, Roslagsgatan 17, SE-11355 Stockholm, Sweden; 6 Department of Geography, Kent State University, Kent, Ohio 44240, USA; 7 Biological Control and Spatial Ecology, Université Libre de Bruxelles, B-1050 Brussels, Belgium; 8 Department of Geography, University of Namur, B-5000 Namur, Belgium; 9 Institute for Health Metrics and Evaluation, University of Washington, Seattle, Washington 98121, USA; 10 Fogarty International Center, National Institutes of Health, Bethesda, Maryland 20892-2220, USA; 11 Wellcome Trust Centre for Human Genetics, University of Oxford, Oxford, OX3 7BN, UK

**Keywords:** Sustainability, Geography, Databases

## Abstract

According to UN forecasts, global population will increase to over 8 billion by 2025, with much of this anticipated population growth expected in urban areas. In China, the scale of urbanization has, and continues to be, unprecedented in terms of magnitude and rate of change. Since the late 1970s, the percentage of Chinese living in urban areas increased from ~18% to over 50%. To quantify these patterns spatially we use time-invariant or temporally-explicit data, including census data for 1990, 2000, and 2010 in an ensemble prediction model. Resulting multi-temporal, gridded population datasets are unique in terms of granularity and extent, providing fine-scale (~100 m) patterns of population distribution for mainland China. For consistency purposes, the Tibet Autonomous Region, Taiwan, and the islands in the South China Sea were excluded. The statistical model and considerations for temporally comparable maps are described, along with the resulting datasets. Final, mainland China population maps for 1990, 2000, and 2010 are freely available as products from the WorldPop Project website and the WorldPop Dataverse Repository.

## Background & Summary

An increasing global population, becoming more concentrated in urbanized regions, is estimated to contribute another 2.5 billion people to the current total by 2050 ^1^. A large part of this urban population growth is in Asia, where it is increasing by 1.5% per year, with the only other region showing >1% per year urban growth being Africa^1^. One of the most significant components of these Asian population trends is China, with an urban population of 54% in 2014 projected to be ~70% by 2030 (ref. 2). Considering the implications of how continued population growth in the coming decades influences the sustainability of urban regions and general human welfare^3^, a better understanding of the spatial patterns of growth is needed. One way to accurately depict spatially-explicit changes in population distribution patterns is through the use of gridded population datasets.

A dasymetric approach is typically used to disaggregate areal census data to smaller spatial units^4^. Over the past couple of decades, a proliferation of more sophisticated techniques highlights the increasing statistical applications and inclusion of G.I.S. and remote sensing data to inform gridded population datasets^5,6^. While such approaches may be applied at a variety of spatial scales^7,8^, the most commonly used global and regional datasets include the WorldPop Project^5^, the *Gridded Population of the World* (GPW)^9,10^, the *Global Rural-Urban Mapping Project* (GRUMP)^11^, *LandScan* ^12^, and the United Nation Environment Programme *East Asia Population Database* ^13^. All of these datasets rely on different approaches, assumptions and input data to generate gridded population outputs at varying spatial resolutions (~3 to 150 arcseconds).

The WorldPop project (www.worldpop.org) provides datasets at the finer end of the spatial spectrum, at 3 arc seconds (~100 m spatial resolution at the equator), for Africa, Asia and Latin America. These are constructed for the year of the input population data and also for 2010, 2015, and 2020, unadjusted and adjusted using urban and rural growth rates taken from the United Nations World Urbanization Prospects Database, 2014, to match UN Population Division national total estimates^1^. The traditional framework of the WorldPop project has enabled the development of a machine-learning based approaches for mapping populations at fine spatial resolutions (i.e., at 3 arc seconds and 100 m) that have been shown to improve on accuracies of previous approaches^5^. The modeling framework is a two-step process that applies a Random Forest-based model to generate a prediction weighting layer subsequently used to inform a gridded dasymetric re-distribution of original census counts^5^.

**Figure 1:**
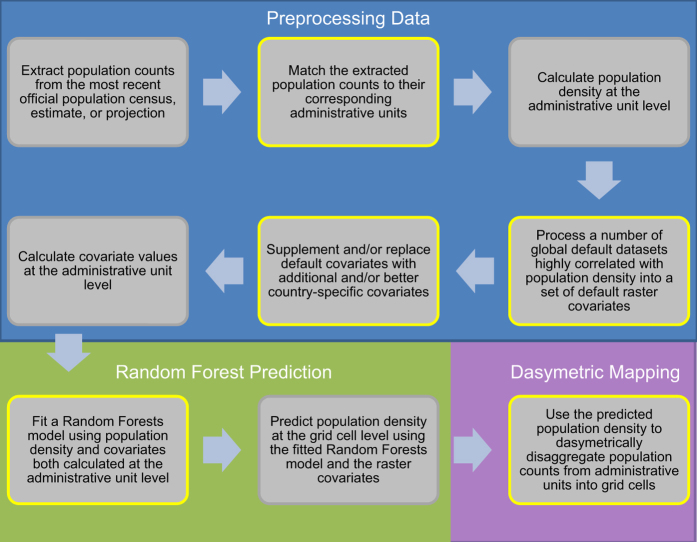
Flow diagram of the WorldPop approach to mapping population. Conceptual overview of the Random Forest-based dasymetric mapping approach used to produce the ‘WorldPop’ datasets including the key steps that involve adjustments to make final population datasets comparable over time (modified from Stevens *et al.* ^5^). Three primary processing stages are highlighted in the blue, green and purple areas of the figure. Steps outlined in yellow are those that are needed for producing temporally-comparable datasets.

In this paper, we describe how the WorldPop Random Forest-based model can be used for analyzing population change. Figure 1 depicts the two-part modeling approach outlined in Stevens *et al.* ^5^, and used by Sorichetta *et al.*,^14^ for modeling population distribution in 26 countries located in Latin American and the Caribbean. Steps outlined in yellow represent parts of the process that demand additional adjustment or attention when constructing the model for temporally-comparable datasets. These considerations are explained in more detail below.

The specific datasets presented are for mainland China. In the past three decades, the scale of urbanization and migration to cities in China has been substantial, with the total urban share of population increasing from 17.9 to 53.7%. Prompted by government policies and economic development, from 1978–2013 the number of cities increased from 193 to 658, and towns from 2173 to 20,113 (refs 2,15). By 2030, China’s urban population is predicted to grow by an additional 310 million people and the fine-scale granularity (i.e., 3 arc seconds and 100 m) of the described population datasets provide a historic baseline of gridded population values for mainland China that will facilitate a better understanding of the growth, shape, and change in population distribution since 1990.

The following sections outline the open-access archive of temporally-comparable, high-resolution datasets of gridded population distribution for mainland China for 1990, 2000, and 2010. To ensure that maps are comparable between years, we incorporate Landsat-derived urban extents for each year, with other time-invariant and temporally-explicit datasets and county-level census data for 1990, 2000, and 2010. The resulting population datasets are the first to show fine-scale, spatially-explicit depictions of mainland Chinese population distribution patterns that have been associated with national policy reforms, which have shifted the economic base, and thus population, to urban areas across China^16^.

## Methods

### Review of the WorldPop Project

The WorldPop project (www.worldpop.org) developed by the authors, creates and maintains a database of contemporary, high resolution global demographic data. It is currently the only provider of open, high resolution (100×100 m) spatial demographic data on population distribution and composition across national and regional scales, built using peer-reviewed methods^5,14,17,18^. With 82% of the World’s population mapped across 166 countries, WorldPop data are widely used by governments, researchers and organizations across the globe. These data are a key component in hundreds of studies where geography is important, particularly those focused on population health, food security, climate change, conflicts and natural disasters^19–25^ – *knowing where populations are and their demographic features forms the basis for accurate assessments of impact.*

### Model construction

#### Data Processing

Prior to model implementation, necessary datasets must be acquired and pre-processed for the country of interest. Chinese population data were obtained from the National Bureau of Statistics of China via the Chinese Center for Disease Control and Prevention at the Quxian level (county level) and joined to their corresponding GIS-census administrative boundaries for 1990 (refs 26,27), 2000 (refs 28–30), and 2010 (refs 31,32). For boundary and data consistency across years, the Tibet Autonomous Region, Taiwan, and the islands in the South China Sea (except for the Hainan Island) were excluded. The total population for each year and the corresponding number of census units are given in Table 1.

**Figure 2:**
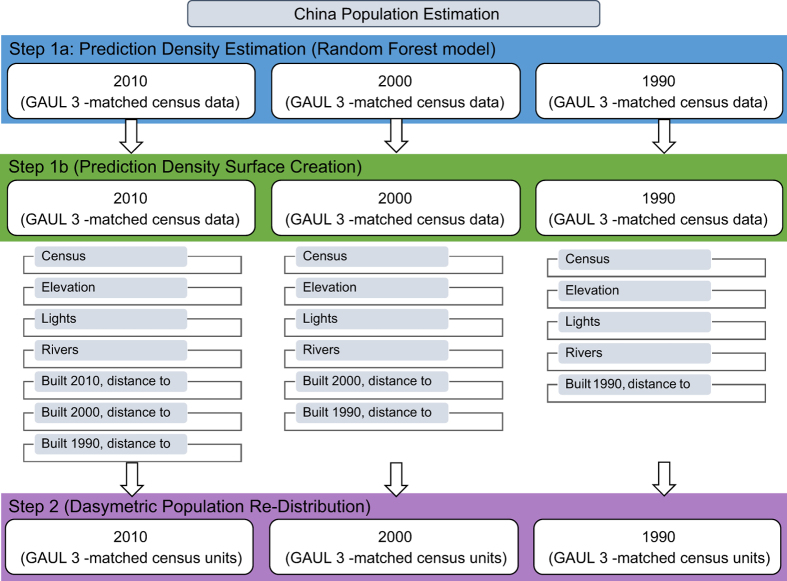
Specific steps for temporally-explicit WorldPop modeling approach. Overview of the modeling process. Temporally-explicit data include census data, DMSP lights at night data, and urban extents by year.

To facilitate comparison of final population datasets, the original census datasets were aggregated to the uniform Global Administrative Unit Layer (GAUL), administrative level three (2,922 units), which are based on the Food and Agricultural Organization framework^34^. Identical census units were desirable to ensure a consistent estimation process across all three years, i.e., reduce over- and under-fitting due to large variations in census unit size and therefore average population densities. Census administrative units falling within a single GAUL unit were assigned completely to that unit, while those falling in more than one had their population count weighted by the area falling inside the respective GAUL units. The standardized boundaries were used for both the Random Forest estimation^5^ and the dasymetric redistribution portions of the mapping (Figure 2).

To produce temporally-comparable datasets this model uses only default covariates that are either time-invariant or temporally explicit (Figure 2). Landsat-derived built land cover extents were acquired for each model year to provide an account of urban development dynamics^19^. A comprehensive overview of that data process is found in Wang *et al.* ^35^ To best use the urban extent information we created a distance-to-built-edge covariate, where distances inside the built land cover class boundary were negative and distances outside the edge were positive. We also used data from the DMSP-OLS (v.4) lights at night time series, obtained from NOAA’s National Geophysical Data Center^36^. Since the time series extends back to 1992 we used that year for the 1990 model. Two satellite datasets were available for 2000 and so we took the average (F14 and F15) for input into the 2000 model. We used the single lights dataset available coincident with 2010. Lastly, we included elevation and its derived slope (source: HydroSHEDS^37^) and distance-to rivers (source: OpenStreetMap^38^) assuming that these variables have not changed dramatically over the past twenty years.

Furthermore, for producing temporally explicit WorldPop models, an additional covariate representing the preceding years’ ‘distance-to-built’ layer(s) was used. The rationale behind including each year as an individual covariate was to ensure the Random Forest algorithm could incorporate changes in settlement/urban extents between years thereby allocating population according to built-area history in addition to the contemporaneous built extent. This approach is intended to provide more nuanced information about development history for these areas specifically with respect to a potential decrease in population density for urban core areas^39^ which contrasts to that where built-area expansion took place.

### Step 1

#### 1a. Prediction Density Estimation

A Random Forest regression^40^ was used to predict population density at the census unit level23. The non-parametric approach is characterized by a flexible and robust framework that allows varying data types to interact with each other in the model. An ensemble decision-tree classifier or predictor, the Random Forest algorithm allows for generation of unpruned decisions trees, essentially growing a ‘forest’ of individual trees which are then aggregated to produce a final predicted estimate^40^.

The predictive capability of the Random Forest model is strengthened by the random selection of predictors at each node in each tree^40,41^, making the final performance comparable to other types of regression trees but requiring fewer set parameters to fit the model^42^. The main parameters that need to be given consideration include (i) the number of covariates needed to randomly select the best covariate for each node during the forest growing process (ii) the total number of trees for each forest, and (iii) the number of observations in the terminal nodes of each tree. In this case, each model used all covariates in the selection process (Figure 2), and each forest had a total of 500 trees and a single observation for each terminal node.

In addition, prediction error at the unit of observation level may be calculated using one-third of the data held in reserve during the iterative ‘bagging’ process for each tree in each forest, which are then used to estimate an ‘out-of-bag’ (OOB) error rate^40^.

#### 1b. Prediction Density Surface Creation

**Figure 3:**
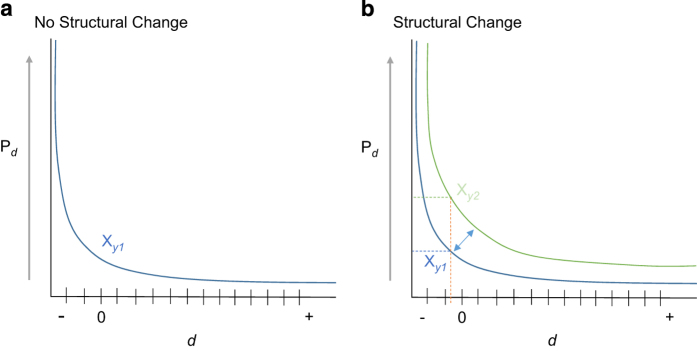
Hypothetical illustrations of the underlying relationship for prediction density (P_*d*_) and distance to built-area-edge (*d*) when there is an assumption that the relationship does not change over time (**a**) and when the relationship does change over time (**b**).

Once the parameters of the prediction density estimation process have been set, a country-wide, 100 m pixel-level map of predicted population density is produced. Considering the immense amount of change across China since 1990, both with respect to population growth and urbanization, each year is estimated independently of the others (Figure 2). We illustrate this need in Figure 3 by depicting the underlying RF relationship for prediction density (P_*d*_) and distance to built-area edge (*d*). Illustrated in panel *a* is an underlying assumption that the relationship between P_*d*_ and *d* does not change over time. If that assumption holds it would be valid to use a RF model parameterized on finer-scale census data from a specific year for others. In contrast, the assumption illustrated in panel *b* shows the more realistic case where the relationship between P_*d*_ and *d* changes over time. In this case where the structural relationship of population distribution with built-area changes, like we know it does in area where rapid urban development may outpace population growth or migration, it would be inappropriate to apply a model that does not incorporate temporality. The simplest way to address the temporality consideration is to fit separate models for each year.

**Figure 4:**
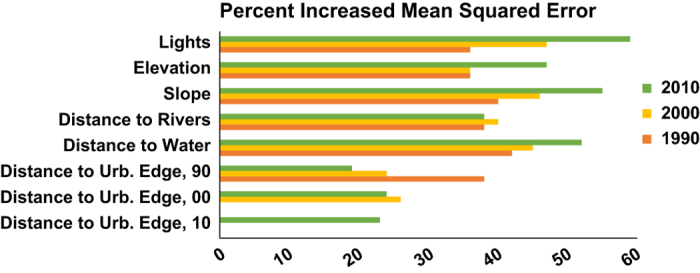
Percent increased mean square error which indicates the variable importance for each year’s Random Forest regression. Variable importance for each year’s Random Forest regression, presented as the percent increased mean squared error when the variable is used but randomly resampled for producing out-of-bag (internally cross-validated) predictions. Each model with representative variables shown by the color bars above were used to produce the density weighting layer for the dasymetrically distributed population map, respectively.

After fitting, each individual model covariate is permuted and OOB estimates are produced using that permuted data. The decrease in prediction accuracy is a robust measure of the ‘importance’ of the permuted covariate to the fit of the final model. The variable importance for each modelled year is highlighted in Figure 4, with higher values of percent increased mean squared error indicating which variables were most important in the OOB cross-validation process.

By examining Figure 4, the importance of the covariate ‘Lights’ is substantial for all three years although it becomes increasingly important in 2000 and even more so in 2010. This may relate to the increasingly urbanized, and subsequently, ‘lit’ regions around the country. In contrast, the ‘Distance to water’ covariate is also an important variable in the model and increases in importance due to increasingly urbanized parts of mainland China along the eastern seaboard, but is the most significant covariate only in 1990. The ‘Distance to Built Edge’ covariates highlight the contribution of existing and increasing urban areas to the redistribution of population across all three years. ‘Elevation’ and ‘Slope’ are important due to the high concentration of the Chinese population in more low-lying regions of the country.

### Step 2cc

#### Dasymetric Population Mapping

**Figure 5:**
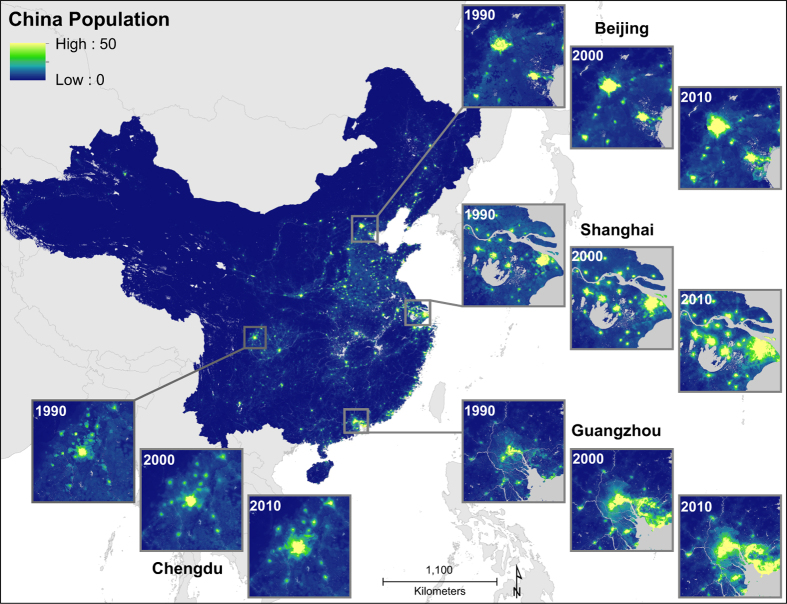
Predicted people per grid cell across mainland China with subsets highlighting the specific years 1990, 2000, and 2010. Estimated people per grid cell across mainland China for 1990, 2000, and 2010 (grid cell resolution is 3 arc seconds, or ~100 meters at the equator). The Tibet Autonomous Region, Taiwan, and the islands in the South China Sea (except for the Hainan Island) were excluded. Projection is in Asia Lambert Conformal and grid cell value represent people per hectare (pph).

The prediction density layer produced by the Random Forest is then used as a weighting layer in a standard dasymetric redistribution approach. The population counts from the boundary-matched GAUL 3 administrative units are disaggregated to 100 m grid cells, producing three gridded population datasets that represent the predicted number of people per hectare for each modeled year. Recall that GAUL 3 units were used since their boundaries are time invariant, and those boundaries are used for all zonal statistical calculations within the dasymetric redistribution process to compare across years. After projecting back to geographic coordinates (datum:WGS84) final end-user products include raster (i.e. gridded) maps of population distributions with a pixel size of 3 arc seconds x 3 arc seconds (~100 m×~100m at the equator) along with people per hectare datasets across all of mainland China, for 1990, 2000 and 2010 (Figure 5).

### Code availability

The Python (version 2.7.5; https://www.python.org/download/releases/2.7.5/) and R (version 2.15.3) programming language scripts used to produce the ‘WorldPop China Mainland’ datasets described in this article are publicly available and can be freely downloaded from *figshare* [Data Citation 1].

## Data Records

The high-resolution, temporally-comparable ‘WorldPop China Mainland’ datasets [Data Citation 2] are stored in the WorldPop Dataverse Repository, and may also be freely accessed from the WorldPop Project website (www.worldpop.org/data/). From the project website the files may be downloaded as a 7-zip file archive (7-Zip.org) or as individual GeoTIFF datasets. Each 7-zip file contains the mainland Chinese population datasets for the respective year, including the estimated people per grid cell as people per hectare and people per pixel (Table 2).

For each year, the predictive covariates used are described in the HTML metadata report that accompanies the corresponding gridded population datasets. The metadata report also illustrates the population density estimates that were used to dasymetrically disaggregate the population from administrative unit to grid cell level, and basic information about the Random Forest model. The prediction error, relative importance of each covariate, and the prediction intervals using the out-of-bag (e.g. mean squared error) data are included in the report.

## Technical Validation

Accuracy assessment of gridded population products was done using a summed gridded population count value, by respective year, compared to a finer-level Jiedao/Xiangzhen (i.e. township) level count for the urban centers of Shanghai, Beijing, Guangdong and Chongqing. The Jiedao/Xiangzhen population data were obtained from the China Data Center at the University of Michigan (http://chinadatacenter.org/). Acquisition of the entire mainland China township-level data was not feasible, and thus, these four central regions were determined to provide the most comprehensive and geographically relevant regions of China for evaluating the results of the population modeling process. While an ideal assessment of the gridded population datasets accuracy would involve a cell-by-cell count comparison the cost and time associated with that type of data collection is difficult. The finer-scale census counts provided by the Jiedao/Xiangzhen level data provide a means to evaluate how well the estimated population from the gridded output compares to population counts summed at the Jiedao/Xiangzhen level.

Two primary statistics are used to describe model performance, root mean square error (RMSE) and mean absolute error (MAE). Each statistical measure provides insight into the accuracy of the final population outputs. We also report RMSE per unit area (RMSE standardized by area) and the percent RMSE (RMSE expressed as a percentage of the total population at the Jiedao/Xiangzhen level). The MAE is less sensitive to outliers in the prediction output than RMSE, with a larger difference between MAE and RMSE indicating greater variance in individual errors. We also calculate the median absolute deviation (MAD) which is another measure robust against outliers and informative when examining population counts or densities whose distributions are highly skewed and where relatively few but very large errors can affect MAE and RMSE disproportionate to their frequency in the data.

### Assessment of gridded population datasets

**Figure 6:**
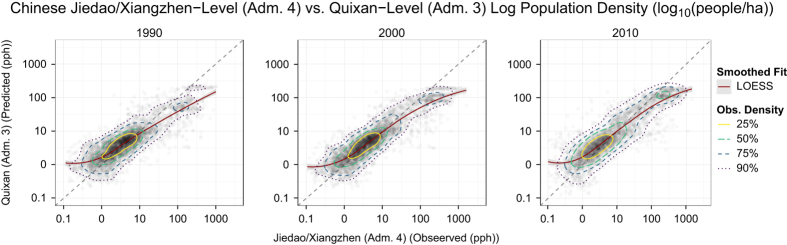
Model fit between the predicted population unit counts at the Jiedao/Xiangzhen unit compared to the original census counts at the same unit level. Comparison of validation unit counts divided by unit area (population density) on a log_10_-log_10_ scale with those estimated from maps produced using coarser census units. Jiedao/Xiangzhen (Admin. Level 4) were used as validation units and estimated population maps were produced using Quixan (Admin. Level 3) data. The Jiedao/Xiangzhen units represent the finest level census data available for the urban centers of Shanghai, Beijing, Guangdong and Chongqing ((4,274 validation units (1990), 4,274 validation units (2000), 3,265 validation units (2010)). This comparison is an estimate of overall model fit at the Admin. Level 4 level. Contours are plotted at observation density thresholds above which the specified percentage of observations are found. The smoothed fit line showing overall trend is estimated by LOESS (Cleveland, *et al.* 1992) (ref. 42) (smoothing parameter alpha=0.75).

**Figure 7:**
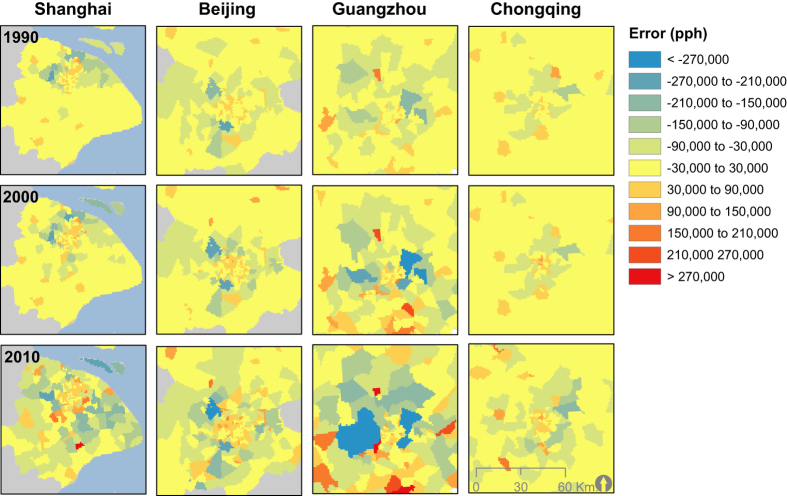
Errors shown in people per hectare based on the validation analysis for year. Errors produced from the validation calculation for each population distribution year for Shanghai, Beijing, Gaungdong and Chongqing. Population underestimates are highlighted in blue and overestimated values are shown in red.

Figure 6 shows the model fit between predicted population unit counts summed up by total number of people inside each Jiedao/Xiangzhen unit compared to the original census counts at the Jiedao/Xiangzhen level for 1990, 2000, and 2010. The number of administrative units contained in the 1990 and 2000 validation data set totaled 4,274 while the 2010 validation data set had a total of 3,265. The distribution census counts for each model suggests a very good fit at low to medium population densities, but with increasing errors at extremely high population densities (Figure 7). At very high population counts, there is greater underestimation of the observed data. This type of error shows that the modeling process does not concentrate people heavily enough in highly urban areas and instead spreads estimations out to less densely populated areas. This is inherent to the dasymmetric approach used in the population redistribution process of the model, but affects relatively few total census units as observed by the marginal frequency histograms in each panel of Figure 6.

The statistical outputs are also summarized in Table 3 along with the same validation calculations using observed and estimated population densities for each Jiedao/Xiangzhen unit. The population density values represent the sum of all people in a census unit divided by the number of pixels from the population map falling within the unit. In effect, the population density comparison controls for the size of the census units and indicate similar patterns in the validation results.

### Assessment of the Random Forest model

The Random Forest model produces the population density weighting layer that is then used in the dasymetric process to redistribute the original census counts within each administrative unit. The variance explained in predicting GAUL 3-based population density observations for each year was 85, 88, and 86% for 1990, 2000, and 2010, respectively. It should be noted that the model fitting process occurs at the administrative unit level and thus the out-of-bag (OOB) prediction error is most appropriately interpreted at the administrative level rather than the grid cell level.

The OOB estimates provide a prediction of the overall model accuracy of the Random Forest estimation process. The process is done by averaging all mean squared errors from 1/3rd of the observations withheld from the iterative bagging process for each individual tree in the forest. The OOB error in predicted GAUL 3-based log population density (mean of squared residuals) for each year is 0.37, 0.30, and 0.35 for 1990, 2000, and 2010 models.

## Usage Notes

Monitoring and mapping population and urban growth is essential for effective planning and resource allocation across the world. Existing datasets and methods traditionally produce single snapshots of population distributions, within limited frameworks that are temporally incomparable. The datasets described here provide timely measuring and mapping of residential mainland Chinese population patterns for 1990, 2000 and 2010, generating comparable datasets suitable for analyzing population change across time. To accomplish this task, the model used a limited set of covariates that were time-invariant or temporally-explicit. This approach supports population density and urban definition change analyses in the most robust and accurate manner available at this time. The datasets can be used in support of identifying and modelling populations at risk in epidemiological, climate, and disaster management applications, among others. In contrast to contemporary WorldPop datasets that use a large ancillary set of data in the modeling process, the reduced level of covariates make these population maps decrease the potential of endogeneity in subsequent analyses.

## Additional Information

**How to cite this article**: Gaughan, A. E. *et al.* Spatiotemporal patterns of population in mainland China, 1990 to 2010. *Sci. Data* 3:160005 doi: 10.1038/sdata.2016.5 (2016).

## Supplementary Material



## Figures and Tables

**Table 1:** Summary information about the original census counts and administrative unit data used to produce the temporally-comparable China population maps

**Year**	**Total Population**	**No. of admin units**	**Avg. Spatial Resolution**	**Admin. Level**	**Data Source**
1990	1,130,822,989	2420	62	Quixan	China CDC
2000	1,242,611,700	2873	57	Quixan	China CDC
2010	1,339,604,009	2925	56	Quixan	China CDC
For each year, the Average Spatial Resolution (ASR)^33^ was calculated as the square root of its surface area divided by the number of administrative units to provide an average measure of the ‘cell’ size of administrative units if all units were squares of equal size).					

**Table 2:** Name (CHN and YEAR represent the China ISO country code and the population count year, respectively), description, and format of all files contained in each 7-Zip file.

**Name**	**Description**	**Format**
CHN_ppp_v2c_1990.tif	Projected estimated people per grid cell for 1990 (3 arc seconds)	GeoTIFF
CHN_pph_v2c_1990.tif	Projected estimated people per hectare for 1990	GeoTIFF
CHN_1990_metadata.html	Metadata report for the Random Forests model	HyperText Markup Language
CHN_ppp_v2c_2000.tif	Projected estimated people per grid cell for 2000 (3 arc seconds)	GeoTIFF
CHN_pph_v2c_2000.tif	Projected estimated people per hectare for 2000	GeoTIFF
CHN_2000_metadata.html	Metadata report for the Random Forests model	HyperText Markup Language
CHN_ppp_v2c_2010.tif	Projected estimated people per grid cell for 2010 (3 arc seconds)	GeoTIFF
CHN_pph_v2c_2010.tif	Projected estimated people per hectare for 2010	GeoTIFF
CHN_2010_metadata.html	Metadata report for the Random Forests model	HyperText Markup Language

**Table 3:** Population Unit Counts and Population Densities statistical metrics produced from the validation calculations for each population distribution year.

	**1990**	**2000**	**2010**
*Population Unit Counts*			
RMSE	24711.91	33051.72	50890.18
RMSE/Area	66.37	91.46	115.12
%RMSE	95.84	96.98	94.52
MAE	15441.75	18608.52	31045.17
MAD	9604.47	9597.7	17231.16

*Population Densities*			
RMSE	66.37	91.46	115.12
%RMSE	258.14	249.53	219.97
MAE	17.29	24.08	34.17
MAD	1.64	1.64	2.32
